# Rationale and study design for decision making & implementation of aging-in-place/long term care plans among older adults

**DOI:** 10.1016/j.conctc.2021.100756

**Published:** 2021-03-16

**Authors:** Lee A. Lindquist, Ruqayyah Muhammad, Amber P. Miller-Winder, Lauren Opsasnick, Kwang-Youn Kim, Julia Yoshino Benavente, Michael Wolf, Vanessa Ramirez-Zohfeld

**Affiliations:** Division of General Internal Medicine and Geriatrics, Feinberg School of Medicine, Northwestern University, Chicago, IL, USA

**Keywords:** Geriatrics, Aging-in-place, Alzheimer's disease, Decision-making

## Abstract

**Background:**

Remaining in one's own home and community is a priority for many older adults as they age. Decision-making and planning is critical to ensure successful aging-in-place (AIP), especially when individuals experience age-related changes such as cognitive decline. *Objectives*: We are testing how decision-making and planning for AIP is impacted by changes in older adults' cognition and function, chronic conditions, social influences, environmental factors and identifying the mediating/moderating interactions between factors. We will also assess whether decision-making and planning for AIP translates into timely adoption of plans and goal concordance between older adults and their surrogate/caregiver decision makers.

**Methods:**

We will conduct a longitudinal single-group interventional clinical trial of community-dwelling older adults who are enrolled in LitCog, (R01AG03611) and expose them to an online intervention, PlanYourLifespan.org, which facilitates decision-making and planning for AIP. Enrolled participants (n = 398) will complete interviews at baseline, one month, and every six months up to 42 months in conjunction with the LitCog study, where cognitive, social, functional, and health literacy data is collected. Additionally, we will collect data on decision-making, resource use, communication of plans, timing of plan implementation, and goal concordance.

**Projected outcomes:**

Findings from this study may generate evidence on how age-related changes in older adults may affect decision-making and implementation in relation to AIP as well as the impact of social relationships and support. Ultimately these findings may help shape the design of programs and practices that may improve the lives of older adults and the capacity of institutions to adapt to societal aging and AIP.

## 1 Introduction

Living in one's own home and community is paramount to many older adults as they age [[1], [2], [3]]. Older adults who remain in their own homes often report greater satisfaction and less depression, and maintain their physical function better than those residing in assisted living or nursing home settings [4]. Over time older adults face increasing frailty and disability, requiring additional support to remain in their homes or placement in long term care facilities [[5], [6], [7]]. The lifetime probability of becoming disabled in at least two activities of daily living or being cognitively impaired is 68% for people age 65 and older yet individuals underestimate the likelihood that they will need assistance in the future [8,9]. Results from the 2012 National Health Interview Survey showed that 60% of older adults believed they were unlikely to need long-term care services in the future, whereas evidence suggests that nearly 70% of older adults will need them at some point.[10,11] Although many older adults will need support [12,13], they may avoid decision-making about their future needs [14]. This lack of decision-making often translates to unsafe living conditions, poor health outcomes, and burden/stress for families.[15,16].

Older adults frequently depend on their families for navigating health crises and care needs [17]. A common problem is that individuals do not communicate their future home care needs and preferences.[18] If critically ill, families often must consider placement in skilled nursing facilities, long term care, hiring caregivers, or become a caregiver themselves.[19,20] With worsening Alzheimer's disease, families often have to make decisions without knowing what their loved one prefers [21].

Age-related changes, such as cognitive decline, likely impact decision-making [22]. Onset of cognitive decline is subtle for most, yet these subtle age-related cognitive changes can detrimentally affect individual decisions that are critical for maintaining health and well-being [[23], [24], [25]]. To effectively make informed decisions, older adults must rely upon a range of cognitive skills to access, use, apply and remember health information and instructions [[22], [23], [24], [25], [26]]. Components of cognition that are involved in decision making include attention (e.g. concentration on the issues for the decision), remote and recent recall (e.g. remembering the historical influences and current events that would impact a decision), executive function (e.g. making the decision), language (e.g. conveying the decision), and abstraction (e.g. connections between decisions and future effects) [[24], [25], [26]]. When the onset of cognitive decline occurs, it can negatively affect the ability to make decision, resulting in life-changing errors. Most studies have neglected the direct mediation between cognitive performance and decision-making; consequently, few interventions have been tested for mitigating the impact of poorer cognitive function on decision-making.

Yet, cognition is only one factor that may impact decision-making in older adults [27]. Health literacy skills also likely play a role [28]. Health literacy, which is more common in older adults, is the degree to which individuals have the capacity to obtain, process, and understand basic health information needed to make appropriate health decisions [28]. Therefore health literacy skills may impact decision-making specific to aging-in-place (AIP)/long term care preferences. Additionally, there are multiple under-studied, mediating/moderating factors that may influence decision-making about AIP and support needs. Social support and influences, such as having involved or influencing family/spouses, friends, or caregivers may impact decision-making [29,30].

To evaluate how decision-making and planning for AIP is impacted by a range of age-related changes in older adults as well as social and environmental factors, we designed a longitudinal, single group interventional clinical trial of participants currently enrolled in the LitCog study who will be exposed to an online in intervention that facilitates decision making, PlanYourLifespan.org [31]. We will also assess whether decision-making and planning for AIP translates into timely adoption of plans and goal concordance between older adults and their surrogate/caregiver decision makers.

### 1.1 Study overview

We are conducting a longitudinal single-group interventional clinical trial of community-dwelling older adults who are currently enrolled in the Health Literacy and Cognitive Function among Older Adults (LitCog) research study (R01AG03611) that involves extensive cognitive testing. Participants that enroll in this study will be exposed to an online intervention, PlanYourLifespan.org (PYL), which facilitates decision-making and planning for AIP. This tool was previously used in a randomized controlled trial (Clinicaltrials.gov Identifier: NCT02256072).

In study aim 1, we will determine how decision-making and planning for AIP is impacted by older adults’ age-related changes (e.g. function, cognition, multiple chronic conditions), social influences (e.g. offspring, spouses), and environments (e.g. rural/urban, home type). We hypothesize that older adults with functional loss, cognition issues, advanced medical complexity/health crises (e.g. hospitalizations) will demonstrate a greater likelihood to complete decision-making for AIP; that older adults with involved families will predict decision-making completion and communication of decisions; and that concerns about personal environments (e.g. living alone in large home, rural access to home support needs) will impact decision-making and planning for AIP. This hypothesis is supported by prior research that has shown that older adults who are healthy tend to delay decision making for AIP until the need arises [14].

In study aim 2, we will examine the mediating and moderating interactions between older adult age-related changes, social influences, and environments in decision making and planning for AIP choices (Fig. 2). We hypothesize that treatment burden, social support, patient activation, and personality will moderate while the decision-making tool (PYL) will mediate longitudinal associations of cognitive function, health literacy, and self-management skills; older adults who have impaired cognition (e.g. mild cognitive impairment, early Alzheimer's), lower health literacy, and struggle with their self-care management will need to make and implement decisions about home based resources/long term planning sooner than those with adequate health literacy and cognition; older adults with high levels of social support (e.g. close family, spouse) will more likely complete decision-making to better communicate wishes and avoid becoming a burden to family members; and that older adults who are healthier with lower treatment burden will demonstrate a lesser likelihood to complete AIP decision-making, when compared to older adults with high treatment burden/chronic conditions.

In study aim 3, we will assess whether decision-making and planning for AIP translates into implementation and goal concordance for older adults and their surrogate/caregiver decision makers. We hypothesize that decision-making and planning for AIP will translate into goal concordance between the older adult's plans and the implementation of those plans.

## 2 Methods

The study protocol was approved by the Northwestern University Institutional Review Board, and is registered on Clinicaltrials.gov (NCT03960476).

### 2.1 Study setting

Study participants will complete in-person research activities (consent, baseline interview, intervention completion, and post-intervention survey) in research space available at Northwestern University locations. Study follow-up surveys will take place over the phone. To adapt to the COVID-19 pandemic, in-person activities will be revised to allow for completion remotely, over the phone.

### 2.2 Study sample

We will recruit study participants from the LITCOG III: Health Literacy and Cognitive Function among Older Adults study (LitCog). LitCog is a longitudinal cohort study, now in its twelfth year of NIH funding, investigating associations between cognitive function and health literacy and how these factors affect performance on a range of common, health self-management tasks. Initially a cross-sectional study (LitCog I), 900 adults ages 55–74 were recruited in 2008 from 8 community-based, primary care practices in Chicago. Participants completed a series of comprehensive cognitive, psychological, social, behavioral, and health assessments (‘T1′ interview), completing follow-up assessments every 3 years (T2, & T3; 78.2% retention rate). All participants have a chronic illness, 77% have two of more, and 60% have new diagnoses post-enrollment. For this study, we will recruit from a pool of 398 eligible study participants that have completed the fourth study time point (T4) with the goal of enrolling at least 300 study participants. The LitCog study aims are to: 1) determine whether older adults’ health literacy skills change over time, 2) evaluate the association between health literacy, cognitive function, and health status among older adults, and 3) identify psychosocial factors that could mediate/moderate associations. The LitCog cohort is followed every 2.5 years for cognitive testing.

Participants are eligible to participate in this study if they meet the following criteria: 1) age 55 or older, 2) are an active participant in the LitCog study (e.g. they have completed day 2 of the T4 time point of that study, 3) speak and read English, and 4) currently use a computer or tablet with internet access. Participants will be excluded if they have previously participated in the Advanced Planning for Home Services for Seniors study (ClinicalTrials.gov Identifier: NCT02256072) which developed and tested the PYL study intervention.

### 2.3 Intervention

**Fig. 1:**
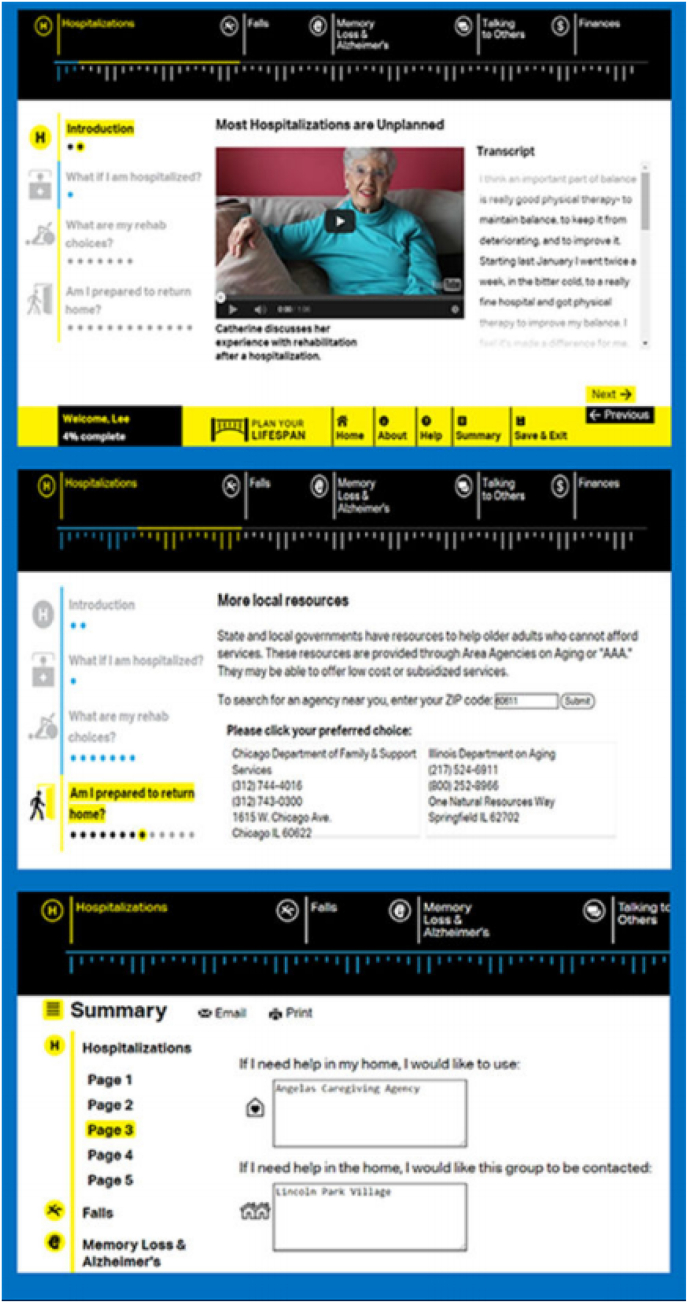
PlanYourLifespan.org intervention.

PlanYourLifespan.org (PYL) is a website that facilitates decision-making and planning for AIP. It focuses on planning and decision-making for health crises that often occur with age and connects users to home-based resources on a local and national level that can be of assistance now or in the future if needed. There are no commercial interests or advertisements in PYL. The content of the website was informed by previous focus groups and the themes include: hospitalizations, falls, memory loss and Alzheimer's disease, as well as sections on communication and financing plans. Each section begins with a video of an older adult (non-actor) discussing their real-life personal experiences of the theme, with subsections providing interactive information on what older adults can expect, types of resources available, and decisions to be made. For example, by entering a zip code, the user can identify the closest Area Agency on Aging or which home caregiver agencies exist in the area (Fig. 1). Users can save their preferences and revisit any decisions made on a variety of topics on the website and can also share their plans/decisions with others by printing or emailing their plans which can stimulate communication about future plans and expectations. As inadequate health literacy and cognitive impairment is prevalent among seniors, PYL presents information understandable at all levels of health literacy and sensitive to cognitive load. [22] The tool uses simplified, large-font, less dense text without scrolling and although it targets people in the United States, other tool information may be useful to seniors from other countries [31,32].

**Fig. 2:**
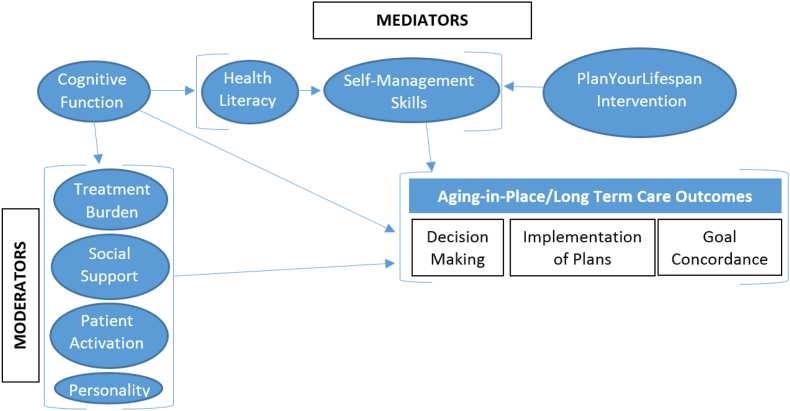
Moderators and mediators conceptual model.

## 3 Procedures

### 3.1 Recruitment

Currently enrolled LitCog study participants who have completed the cognitive testing at LitCog T4 (N = 398) will be invited to participate in this study, as the targeted enrollment. Participants will be mailed a letter informing them about the new study opportunity. Research coordinators will then contact the participants who have not opted out of the study to discuss the study further and complete the eligibility screener. Interested participants who meet the study criteria will schedule a time for the baseline interview which will occur in research space available at Northwestern University locations or remotely by phone during the COVID-19 pandemic.

### 3.2 Study procedures

Prior to beginning the baseline interview, consent will be obtained from study participants using procedures approved by the Institutional Review Board of Northwestern University. Research staff will then administer the baseline study survey either in person or over the phone. Next, they will introduce participants to the intervention, PlanYourLifespan.org, and provide instructions on its use. Research staff can assist with questions as needed on navigation of the intervention but will not assist with decision-making or any content-related questions. A minimum of 15 min and a maximum of 45 min will be allotted for navigating the website. After completing the time allotted on PlanYourLifespan.org, participants will complete a post-intervention survey.

Study participants will be followed up for a total of 42 months, with a total of 8 follow-up study surveys. Research coordinators will contact participants to complete a follow-up survey one month after the baseline interview, at six-months after the baseline interview, and then every six months thereafter. All follow-up surveys will be administered over the phone by research coordinators. Phone follow-up surveys will have a targeted completion window. For the one-month phone follow-up survey, study participants will start to be contacted ±1 week from the date of their completed baseline interview. For the subsequent follow-up interviews, study participants will be contacted ±2 weeks from the date of their completed baseline interview to the study time point. Participants will be compensated financially for completion of the research study interviews as specified on the study consent form.

### 3.3 Data collection

Study data will come from multiple sources and includes: 1) self-reported data by the study participant, 2) data that has been previously collected from the study participants through their participation in the LitCog study (e.g. cognition data), 3) data retrieved from the participant's electronic health record, and 4) data from Google Analytics. All of the electronic health records data we plan to analyze from this study will already be collected for the LitCog study. No additional electronic health records data will be accessed for this study.

We will collect a wide range of measurements such as process outcomes, cognition variables, functional and health variables, and social/environmental variables. Additionally we will collect sociodemographic variables, such as race and ethnicity, an assessment of PlanYourLifespan.org, completion of decision-making using Google Analytics, and timing and goal concordance. Study data are collected and managed using a Research Electronic Data Capture (REDCap) database, a secure, web-based application designed to support data capture for research studies [27].

### 3.4 Participant outcomes

Standard demographic information collected in previous interviews will be used (e.g. date of birth, gender, race/ethnicity, country of origin). As socioeconomic variables may change over time, we will reassess education, household income, marital status, living situation, occupation/employment, health insurance.

Measures of the Aging-in-Place Decision Making Process:

#### 3.4.1 Decision making behavior and communication

We will measure aging-in-place planning and decision making completion using the APHS Planning Behavior and Communication Score, which was previously used in our PCORI-funded study. It assesses beliefs and behaviors towards decision making and communication to others about aging-in-place decisions. With Google Analytics, we will also assess decision making completion, decision changes and timing of decision changes.

#### 3.4.2 Health Services use

Aging-in-place decision making is an ongoing process to make decisions and match resources to patient preferences. While measuring timing of decisions, we will also measure the resources being utilized. The Resource Use Inventory (RUI) is an instrument designed to capture resource utilization and cost in patients with Alzheimer's disease, but has been validated in a sample of community dwelling, cognitively intact, elderly individuals.^113^ The RUI covers use of medical care (e.g. hospitalizations) and non-medical and informal care (e.g. home health aides, unpaid assistance with ADLs, and labor force participation). Outpatient care refers to clinical preventive services, routine and specialty clinic visits, as well as urgent care and ED visits. Inpatient care includes hospitalizations, inpatient rehabilitation (IRF) or skilled nursing facility (SNF) care, and long-term care in nursing homes and continuing care facilities.

#### 3.4.3 Timing and goal concordance between plans and events

t is critical to measure whether these decisions translate to older adult goal concordance and when they occur. We will measure timing and goal concordance on aging-in-place decisions made through PYL. If subjects are hospitalized or fall, they will be asked whether post-hospitalization discharge destinations or support modalities match their preferences. If experiencing worsening memory loss, subjects will be asked whether decisions on aging-in-place, driving, long term care, caregiver assistance matched preferences. These questions will be asked every interview.

Specific mediating and moderating factors in aging-in-place decision making are in Table 1.

**Table 1:** Outcomes and measures.

	MEASURES	DETAILS	LITCOG Collected
Aging-in-Place/Long Term Care PROCESS OUTCOMES			
Contemplation of Decision: Hospitalizations	Participant's thoughts about issues related to hospitalizations	Non-validated, Likert scale. Modified ACP questions.	
**Decision-Making:** **Hospitalizations**	Decision making specifically related to hospitalizations	Non-validated, open-ended	
	Google Analytics	Documentation of decision-making	
**Contemplation of Decision:** **AD & Memory Loss**	Participant's thoughts about issues related to AD & memory loss.	Non-validated, Likert scale. Modified ACP questions.	
**Decision-Making:** **AD & Memory Loss**	Decision making specifically related to AD & memory loss.	Non-validated binary & open-ended questions	
	Google Analytics	Documentation of decision-making	
**Implementation of Plans: Healthcare Utilization**	Hospitalization + Rehabilitation; Emergency Department Visit; Urgent care visits (RUI); Medical Supplies (RUI); Physical therapy (RUI); Long Term Care Facility Use	Individual items, Sano, 2006	X
**Goal Concordance**	Decision made - Outcome concordance	Non-validated binary and open-ended questions	
**Website Satisfaction Assessment**	Perception of the PYL program, satisfaction.	Likert Scale	
**AGE-RELATED CHANGES - COGNITIVE VARIABLES (Mediators)**			
**General Cognition**	Mini-Mental State Exam	Folstein, 1975	X
**Processing Speed**	Digit Comparison; Pattern Comparison; Symbol Digit Modalities	Salthouse, 1992; West Psychology, 1991	X
**Working Memory**	Spatial Span Length - reverse; Spatial Working memory; Size Judgement Span	CANTAB; Cherry, 1993	X
**Inductive Reasoning**	ETS Letter Sets; Ravens Progressive Matrices; Stockings of Cambridge (SOC)	ETS, 1976; Raven, 1976; CANTAB	X
**Long Term Memory**	Immediate Verbal memory; Delayed Verbal memory; New York Paragraph	CANTAB; Kluger, 1999	X
**Verbal Ability**	AM-NART, Graded Naming Test (GNT), Shipley Institute of Living Scale	Grober, 1991, CANTAB, West Psychology, 1986	X
**Health Literacy**	Newest Vital Sign (NVS), Test of Functional Health Literacy (TOFHLA), Rapid Estimate of Literacy in Medicine (REALM)	Weiss, 2005, Parker, 1995, Davis, 1993	X
**AGE- RELATED CHANGES – FUNCTIONAL and HEALTH VARIABLES (Moderators)**			
**Physical Function**	PROMIS (Physical Function - Short Form 10a)	Cella, 2010	X
**Psychological Factors**	Patient Activation Measure (PAM) (13-item); NEO Five Factor Inventory - 3 (NEO–FFI–3); CHAI; IPIP; SAPA (SPI-35, short IPIP)	Hibbard, 2005; PAR; Goldberg, 1992; Condon, 2016	X
**Health Status**	Chronic Conditions; General Health	individual items	X
	Depression (PROMIS Depression - Short Form 8 b) Anxiety; PROMIS (Anxiety - Short Form 7a)	Cella, 2010^158^	X
**Previous Health Experiences: Self & Others**	Participant's past health experiences with self, others.	Non-validated binary & open-ended questions.	
**Medication**	Prescription Medications	chart review	X
	Number of Prescription Medications	individual item	X
**Risk Behaviors**	Smoking Status (Current, Former, Never), Pack Years; Alcohol Use; Berkeley Fat Intake	individual item, BRFSS Block, 2000	X
**Self-Care Complexity**	Healthcare Task Difficulty (HCTD); Medication Regimen Complexity Index (MRCI)	Boyd, 2014	X
		George, 2004	X
**Self-Manage Skills**	Comprehensive Activities Scale (CHAS)	Curtis, 2015	X
**SOCIAL and ENVIRONMENTAL VARIABLES (Moderators)**			
**Social Support**	Lubben Social Network Scale; Social Support – Tangible; Paid/Unpaid Caregiver Assistance	Lubben 20061^59^; Woloshin, 1997	X
**Social Integration**	Support Satisfaction: instrumental; information; emotional	Sander, 1999160	
**General Self-Efficacy**	PROMIS: General Self-Efficacy	PROMIS Self-Efficacy	
**Self-Efficacy for Managing Social Interactions**	PROMIS: Self-Efficacy for Managing Social Interactions	PROMIS Short Form 4a	
**Social Isolation**	PROMIS: Social Isolation *S*–F	PROMIS Social Isolation – Short Form 8a	
**Familism**	Familism Scale – Perceptions of support from family/friends	Angel, 2003^161^	
**Health Info Seeking**	HINTS - Technology Use and Health Communication	Nelson, 20041^62^	X
**Environmental Demands**	Busyness and Routine Scale	Martin 2003	X
**Living Situation**	Residence details, co-occupants, concerns	Non-validated	
**Prior Advanced Care Planning & Perception of Needs**	Living Will, Power of Attorney, POLST	individual item	
	Perception of advanced care planning needs	individual item	

## 4 Analyses

### 4.1 Statistical analyses

For Aim 1 we will model the association between decision-making and planning for AIP and the predictors for age-related changes (e.g. cognition, health literacy, multi-chronic conditions), social influences (e.g. adult offspring, spouses), and environments (e.g. rural/urban, home type) using generalized linear mixed-effects models (GLMM). GLMM are well fit for modeling longitudinal data by allowing us to incorporate random effects, which account for correlated data that may arise from collecting repeated measurements on participants over multiple time points. Additionally, the model uses all available data and does not require that all participants have complete data.

To test for moderation in Aim 2, interaction (or effect moderation) terms between cognitive function, health literacy, and self-management skills with each of the potential moderators will be added separately to each model. Use of PlanYourLifespan.org will also be tested as a mediator for AIP decisions. To test for mediation, we must show: 1) the independent variable (cognitive function and its decline) predicts the potential mediator, 2) the independent variable predicts the dependent variable (AIP decisions), and 3) the addition of the potential mediator eliminates or decreases the strength of the relationship between the independent and dependent variables. If this model holds for a particular mediator, we will proceed by adding it to respective models, where significant relationships between cognition and AIP decisions are found. Cognitive function coefficients will then be examined to determine whether the addition of mediators eliminates or decreases the strength of the relationship.

Structural Equation Modeling (SEM) will be performed to model relationships between cognitive function, health literacy, self-management skills, and AIP outcomes (decision-making, implementation, goal concordance) along with various mediating and/or moderating variables. SEM follows 5 steps: 1) model specification, 2) model identification, 3) estimation of the model, 4) testing model fit, and 5) re-specification (if needed). It is particularly useful when observed variables contain measurement error, are interdependent, and when potentially important explanatory variables (e.g. explicit resource knowledge, experiences) are not included in a model. We predict that latent constructs of health literacy, self-management skills, and usage of PlanYourLifespan.org will mediate relationships between cognitive function and AIP outcomes. In addition, treatment burden, social support, patient activation, and personality will moderate these associations. We are proposing to model change in these latent constructs over the 42 months in a multi-wave model. At each time point, we will model the mean level as well as the change (slope) in level of the latent variables. Growth curve parameters will be modeled similarly across all sets of constructs.

To study how decision-making and planning for AIP translate into timely adoption and goal concordance in study aim 3, we will fit a Cox proportional hazards regression model to test the association between these variables while adjusting for other time-dependent and fixed covariate.

### 4.2 Power calculation

Sample size considerations were based on anticipated confidence interval width for correlation coefficients (and/or partial correlation coefficients to measure mediation effects). We will attempt recruitment of the 398 subjects currently enrolled in LitCog cohort. If only 300 participants are recruited (25% refusal rate), the width of the 95% confidence interval around correlation coefficients will be ~0.20 with >95% power to detect a significant (nonzero) sample correlation, assuming a Type 1 error rate of 5%. Using change in health status as a substitute for decision implementation, correlations between cognitive function and change in health status from LitCog T1 to T2 range (time points from earlier in the longitudinal study) from 0.09 to 0.13 depending on the measure, and we expect these correlations to be stronger with longer follow-up time (~r = 0.2). Given this assumption, a sample of 300 achieves 94% power to detect a non-zero correlation between change scores.

## 5 Discussion

Our study will evaluate how decision-making and planning for AIP is impacted by changes in older adults’ cognition and function, chronic conditions, social influences, environmental factors and identifying the mediating/moderating interactions between factors. We also will assess whether decision-making and planning for AIP translates into timely adoption of plans and goal concordance between older adults and their surrogate/caregiver decision makers.

To our knowledge, this trial is the first to extensively examine AIP decision-making, implementation, and goal concordance of long term plans. The planning and decision-making process will be examined in detail providing insight into how decisions are made, whether older adults create plans and whether or not those plans are implemented as planned.

This proposal will leverage an NIA funded cohort, LitCog, with extensive longitudinal data collection and a diverse sample (by race/ethnicity, socioeconomic status, and chronic conditions) recruited from multiple community sites. Participants were intentionally recruited at a younger initial age (M = 63 years) to allow for capture of onset of cognitive decline, incidence of chronic disease, and adjustment to new patient roles. Per the IOM 2015 cognitive aging report, LitCog is unique in that it offers detailed measurement of older adults' health literacy, cognition, performance on ‘real world’ self-care tasks, with over 10 years of follow-up. This allows us to examine effects of increased disease/treatment burden, cognition, health literacy, self-management skills, and health services use in conjunction with decision making and aging-in-place. LitCog will contain one of the most extensive health literacy datasets with the longest follow-up time. Decline in health literacy in conjunction with decision making towards aging in place has never been studied. With many implicated but under-studied variables related to health literacy pathways, we will advance our understanding of health literacy and decision making with real world aging-in-place ramifications.

Findings from this study may generate evidence on how age-related changes in older adults such as changes in cognition, health literacy, and environment may affect decision-making and implementation of plans in relation to AIP, as well as the impact of social relationships and support. Ultimately these findings may help shape the design of programs and geriatrics practices that may improve the lives of older adults and the capacity of institutions to adapt to societal aging and AIP.

This study is the first research to extensively examine aging-in-place decision making, implementation, and goal concordance of long term plans. The proposal is innovative in using the PYL intervention to facilitate aging-in-place planning and longitudinally observing how PYL leads to goal concordance. To our knowledge, PlanYourLifespan is the only web-based tool available that educates about aging-in-place decisions and connects users to available resources. Findings from this project may potentially inform geriatrics practice and facilitate aging-in-place decisions.

This study will generate needed evidence on how age-related changes in older adults affect decision making and implementation towards aging-in-place, with detailed characterization of the affective, cognitive, social, and motivational parameters involved in decision making. It will provide needed information on how moderation of social (offspring, caregivers, support), environmental, and personal factors exerts effects on both the ability to make and implement aging-in-place decisions. Since this study longitudinally overlays already established lead-in cognitive/social/health data collection from the longstanding LitCog cohort, it will be able to examine: 1.) how changes in cognition/social/health/environment affect aging-in-place decisions, 2.) whether changes in cognition enhance or undermine decision making in aging, and 3.) how shifts in social goals and changes in social relationships and contexts impact the extent to which inter-personal processes affect decision making.

## Funding sources

This study is funded through 10.13039/100000049NIA R01AG05877. Dr Lindquist also receives funding through the Claude D. Pepper Older Americans Independence Center (OAIC) at 10.13039/100007059Northwestern University [10.13039/100000049NIA P30AG059988]. REDCap is supported at the 10.13039/100008250Feinberg School of Medicine by the 10.13039/100007059Northwestern University Clinical and Translational Science (NUCATS) Institute. The content is solely the responsibility of the authors and does not necessarily represent the official views of the National Institute of Aging and/or National Institutes of Health.

## Trial registration

ClinicalTrials.gov Identifier: NCT03960476.

## Declaration of competing interest

All authors: No reported conflicts.
